# Assessing the efficacy of systemic antibiotics for biofilm-associated infection in an ovine model of simulated fracture-related infection

**DOI:** 10.5194/jbji-10-511-2025

**Published:** 2025-12-02

**Authors:** Robert Falconer, David Rothberg, Walker Kay, Connor Hunt, Richard Tyler Epperson, Brooke Kawaguchi, Nicholas Ashton, Dustin Williams

**Affiliations:** 1 Department of Orthopaedics, University of Utah, Salt Lake City, UT, USA; 2 Department of Biomedical Engineering, University of Utah, Salt Lake City, UT, USA; 3 Department of Pathology, University of Utah, Salt Lake City, UT, USA; 4 Department of Physical Medicine and Rehabilitation, Uniformed Services University, Bethesda, MD, USA; 5 Noorda College of Osteopathic Medicine, Provo, UT, USA; 6 School of Medicine, West Virginia University, Morgantown, WV, USA

## Abstract

**Introduction**: Infection remains a major complication of open fractures, with rates reaching up to 70 % after severe injury. Systemic antibiotics often fail to achieve the therapeutic levels needed to disrupt biofilm at the wound site due to compromised blood flow and systemic dilution. This study investigates the efficacy of systemic antibiotics against *Staphylococcus aureus* and *Pseudomonas aeruginosa* monomicrobial biofilms in an ovine model of simulated fracture-related infection (FRI). **Methods**: An established model of long-bone FRI in the right hind limb of mature Rambouillet sheep was adapted. Local soft tissue trauma was induced, the periosteum was stripped from the tibial surface, and a simulated fracture was created on the bone surface. The site was inoculated with mature biofilm grown on fracture fixation plates. Sheep were assigned to a treatment group receiving 10 d of systemic antibiotic therapy or a positive control group that received no treatment. All animals were sacrificed at 21 d, and microbiological and histological analysis was performed. **Results**: Systemic antibiotics failed to produce a statistically significant reduction in *S. aureus* biofilm compared to the positive control. Systemic therapy significantly reduced *P. aeruginosa* bioburden compared to the positive control, but levels remained above the clinical threshold for infection. The histological analysis revealed moderate improvement from systemic treatment. **Conclusions**: This investigation established the limitations of systemic antibiotic therapy in this model of long-bone FRI against *S. aureus* and *P. aeruginosa* biofilms. Microbiological and histological analyses revealed hallmark features of recalcitrance to systemic treatment, validating the utility of this model to study anti-infective therapies. These findings highlight the need for new antibiotic delivery strategies to manage biofilm-associated infections.

## 1 Introduction

Fracture-related infection (FRI) rates have historically shown substantial variability, reaching up to 52 % in the most severe cases of open fracture (Gustilo and Anderson, 1976). Despite advancements in orthopaedic surgery, orthoplastics, antibiotic administration, and infection management, severe fractures in the modern era still become infected 16 %–69 % of the time (Hu et al., 2020; Papakostidis et al., 2011). Systemic antibiotic therapy fails to manage persistent infection for two primary reasons: insufficient antibiotic serum levels that fail to effectively kill biofilm-dwelling bacteria and compromised vasculature that fails to deliver systemic therapeutics to the necessary target site.

An open injury is a pathway for bacterial contamination from the environment or the patient's own microflora (Gustilo and Anderson, 1976). Opportunistic pathogens that live in these natural ecosystems typically reside in the biofilm phenotype: robust, structured communities that differ from planktonic “free-soldier” bacteria and act as armoured fortresses to evade host defences and antibiotic therapy. Encased in an extracellular polymeric matrix, biofilm bacteria exhibit altered metabolism and reduced replication, making them highly tolerant to antibiotics (Li et al., 2020; Singh et al., 2022). Furthermore, sessile bacteria deep within the biofilm downregulate metabolism and division, rendering many antibiotics that target rapidly dividing cells ineffective (Berlanga and Guerrero, 2016; Kapoor et al., 2017).

Biofilm-associated resilience presents a major clinical challenge in trauma care, as bacteria can readily tolerate standard therapeutic doses delivered systemically. Post-operative infection management relies heavily on extended durations of systemic antibiotics guided by culture-based susceptibility testing. However, biofilms that form on bone (sequestra), hardware, and/or necrotic tissue undergo a phenotypic transformation that enhances survival (Costerton et al., 1999). Consequently, eradicating these infections often requires antibiotic concentrations tens to hundreds of times greater than those needed for planktonic cells (Ashton and Williams, 2019). These challenges have previously been demonstrated in this ovine model of FRI (Somawardana et al., 2024; Williams et al., 2025). Against methicillin-resistant *Staphylococcus aureus* (MRSA) biofilms, we found that 10 d of systemic intravenous (IV) vancomycin failed to reduce bacterial load below clinically significant thresholds (10^5^ colony-forming units (CFUs) per gram of tissue) and demonstrated histological outcomes indicating persistent infection (Williams et al., 2025).

Building on these findings, we expanded our investigation to include *S. aureus* and *Pseudomonas aeruginosa*, two of the most commonly isolated biofilm-forming pathogens in FRIs (Corrigan et al., 2022; Depypere et al., 2022; Patel, 2023). This study aimed to establish the efficacy of clinically relevant systemic antibiotic dosing regimens when *S. aureus* or *P. aeruginosa* biofilms were used as initial inocula. Standard treatment for *S. aureus* infections typically involves a parenteral β -lactam or cephalosporin, often combined with oral rifampin when orthopaedic hardware is present (Depypere et al., 2020; Gilbert et al., 2015). *P. aeruginosa* infections are similarly managed with a parenteral cephalosporin or oral quinolone, plus oral rifampin (Gilbert et al., 2015). Rifampin was included in both regimens due to its unique activity against biofilm-embedded bacteria and clinical prevalence in managing biofilm-related orthopaedic infection (Zimmerli et al., 1998). Cefazolin and cefepime were selected in this study as recommended parenteral agents (Depypere et al., 2020).

A 10 d course of systemic antibiotics was selected to parallel prior studies, where meaningful reductions in CFUs and histological signs of remodelling were demonstrated from some therapies in the presence of biofilm-associated infection. This timeline has also proven useful in determining whether systemic antibiotics fail to clear infection, as persistent bacterial burden and histological evidence of infection are typically still evident within this window. In our previous work, a 21 d endpoint following a 10 d course of systemic therapy allowed sufficient time for residual bacteria to proliferate and for histological features of infection to become apparent (Williams et al., 2025). This approach aligns with clinical observations that demonstrate biofilm-associated infections often relapse or persist despite appropriately selected systemic antibiotics (Li et al., 2019; Prada et al., 2021). We hypothesized that after 10 d of systemic therapy, *S. aureus* and *P. aeruginosa* biofilms would remain above the commonly used $10^{5}$ CFU g^−1^ “rule of thumb” for infection and exhibit persistent histological signs of infection (Raahave et al., 1986; Robson and Heggers, 1969).

## 2 Methods

### 2.1 Isolate selection and biofilm growth

*S. aureus* ATCC 6538 and *P. aeruginosa* ATCC 27853 biofilms were grown on 1.75 cm × 1.75 cm × 0.85 cm grade 5 titanium simulated fracture fixation plates using a modified CDC biofilm reactor as previously described (Kay et al., 2022; Williams et al., 2025).

### 2.2 Surgical preparation

A total of 24 male (wether) and female Rambouillet sheep aged 1–3 years and weighing ∼ 50–70 kg underwent surgery. Surgeries were performed with approval and oversight from the Institutional Animal Care and Use Committee (IACUC) at the University of Utah and from the United States Army Medical Research and Development Command's Animal Care and Use Review Office (ACURO). Each sheep was treated with their first dose of rifampin and cefazolin or cefepime, depending on the group (Table 1 “Animal treatment groups”), 30–120 min before surgery.

After the sheep was anesthetized, the surgical limb (right hind) was draped with a protective cloth, hearing protection was inserted into the animal's ear, and the limb was exposed to a pressurized air blast as described previously to simulate soft tissue trauma that is characteristic of fracture injuries. The blast was performed using a Martin Engineering Tornado Air Cannon (Williams et al., 2025, 2019). The sheep was transferred to the operating table, and the right hind limb underwent a presurgical scrub.

### 2.3 Surgical procedure

**Figure 1:**
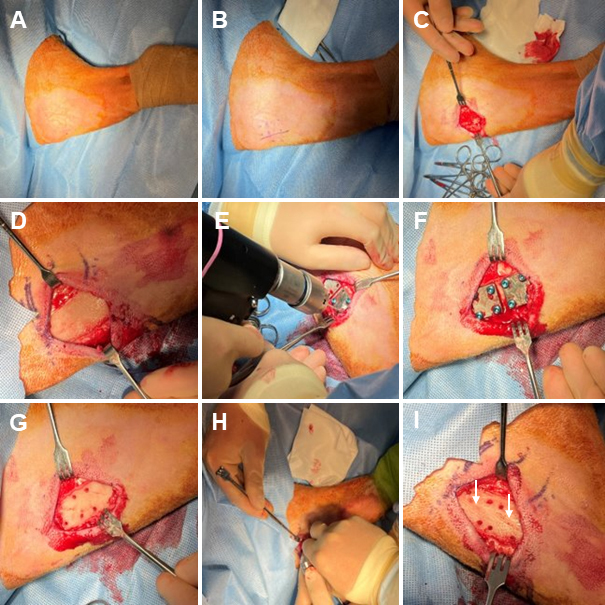
Representative photos of the surgical procedure. **(A)** Sterile Coban is wrapped around the distal leg, and sterile drapes surround the surgical site. The skin is prepped with a povidone-iodine and alcohol presurgical skin preparation. **(B)** The incision line and fixation plate location are marked ∼ 5 cm distal to the tibial tuberosity. **(C)** A 5 cm incision is made parallel to the anterior margin of the tibia. **(D)** Skin, subcutaneous tissue, and the periosteum are removed from the bone surface. **(E)** Template plate areas are drilled to mark the location where the biofilm-ridden fixation plates will reside. **(F)** An image of the template plates secured with cortical bone screws. **(G)** The template plates and cortical bone screws are removed. **(H)** A modified bone saw is used to create a simulated fracture in the area under each plate at a depth of ∼ 2 mm and at an angle of ∼ 20° from the axial direction along the host bone. **(I)** An image of the completed simulated fractures, which are indicated by white arrows.

The surgical procedure was performed as described (Williams et al., 2025, 2012b). A representative image can be seen in Fig. 1. Briefly, an incision was made in the anterior region of the proximal medial aspect of the right hind tibia, the skin and associated subcutaneous tissue were separated from the bone surface, simulated fractures were created on the bone surface, and biofilm-ridden simulated fracture fixation plates were secured to the bone using cortical bone screws.

### 2.4 Animal treatment groups

Animals in the study were randomly organized into one of four treatment groups (Table 1). All sheep received the same surgical procedure.

**Table 1:** Animal treatment groups. The endpoint represents the amount of time since the initial surgery.

Animal group	Bacteria	Treatment	Endpoint
Group 1 ( n=4 )	*S. aureus*	None	21 d
Group 2 ( n=4 )	*P. aeruginosa*	None	21 d
Group 3 ( n=8 )	*S. aureus*	1 g cefazolin IM BID for 10 d 600 mg rifampin PO QD for 10 d	21 d
Group 4 ( n=8 )	*P. aeruginosa*	2 g cefepime IM BID for 10 d 600 mg rifampin PO QD for 10 d	21 d

### 2.5 Post-procedural monitoring and surgical follow-up

The veterinary and research team members remained with the animals after each procedure until they were standing, weight-bearing, and eating. Animals were monitored at least twice daily for signs of pain or distress throughout the monitoring period.

### 2.6 Bone labelling

Fluorochrome labelling techniques have previously been used in ovine models to demonstrate bone formation dynamics (Williams et al., 2025). Animals received an IV injection of calcein green (9.9 mg kg^−1^) via the jugular vein or a forelimb catheter, 5 and 16 d before sacrifice, per published protocols (Epperson et al., 2021; Williams et al., 2012a, b).

### 2.7 Euthanasia and sample harvesting

All animals were euthanized 21 d post-surgery with an overdose IV injection of a pentobarbital-based solution (1 mL/4.5 kg). A veterinary technician or trained lab member verified euthanasia with a vital sign check, i.e. heartbeat, eye reflex, and breathing. The animal's right hind (surgical) limb was disarticulated at the coxofemoral joint by excising soft tissue with a scalpel.

**Figure 2:**
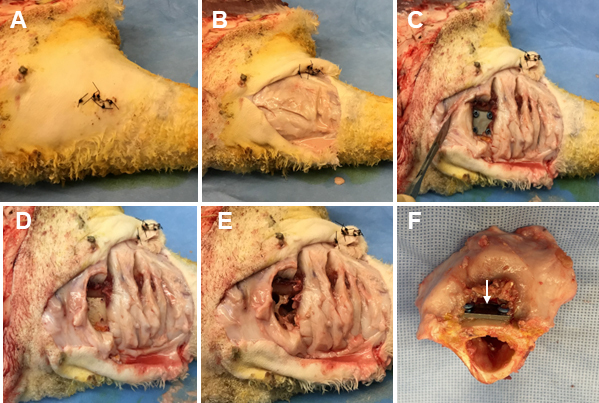
Representative photos of the necropsy sample collection. **(A)** The skin is cleansed using a presurgical skin preparation technique with three alternating scrubs of povidone-iodine and alcohol. **(B)** The original surgical incision is reopened using sterile techniques, exposing the underlying tissue. **(C)** The tissue directly under the distal fixation plate is collected, exposing the plate and cortical bone screws. **(D)** A torque meter (not pictured) with sterilized hex bits is used to remove the cortical bone screws and the fixation plate. **(E)** A surgical drill with sterile trephines is used to remove the cortical bone surrounding each screw track and a section of bone directly under the fixation plate. **(F)** The sectioned bone containing the proximal fracture fixation plate (indicated by a white arrow).

All elements of the microbiological sample collection were performed by a lab member donning sterile gloves using an aseptic technique as previously described (Williams et al., 2025). Samples collected included a tissue section directly above the distal fixation plate, the distal simulated fracture fixation plate and associated screws, a bone section in the centre of the area under the distal plate, the bone surrounding each screw track, and the bone marrow directly under the section of drilled bone. Photographs were taken before and throughout the necropsy (Fig. 2).

### 2.8 Sample quantification

All samples were placed into pre-weighed conical tubes. Tissue samples were transferred to a small Ninja blender and homogenized without overheating. The conical tubes were vortexed for 1 min, sonicated at >40 kHz for 10 min, and vortexed again for 1 min. 100 µ L of the sample mixture was pipetted from the conical tube and immediately plated on a TSA plate as a “0” dilution. Samples were quantified thereafter using a 10-fold dilution series. 50 µ L of the final dilution tube was plated on TSA; the 50 µ L was dispersed in 5 × 10 µ L spots. Plates were incubated at 37 °C for 24–48 h, and colonies were counted to calculate CFU per gram or CFU per sample.

### 2.9 Histological methods

Soft tissues surrounding the proximal simulated fracture fixation plate were dissected away, leaving ∼ 1 cm of soft tissue around the plate. An oscillating bone saw was used to cut the bone down to a smaller section, and the sample was fixed in 10 % neutral buffered formalin with three changes of solution every 48 h. The sample was placed in 70 % ethanol until further processing. μ -CT images were captured and used to create three-dimensional (3D) reconstructions of the bone surface directly underneath the proximal simulated fracture fixation plate (90 kV, 88 µ A, 36 mm field of view, high-resolution scan for 4 min with a Cu 0.1 mm X-ray filter). Before imaging, the two distal screws were removed from the proximal plate to reduce artefacts. The topographical analysis was completed by selecting a 10 × 10 × 1.5 mm region of interest (ROI) centred on the bone under the proximal fracture fixation plate. Additionally, two smaller ROIs (4 × 4 × 1.5 mm) adjacent to the osteotomy were analysed to quantify the percentage of bone post-treatment or post-infection.

After μ -CT analysis, the sample was dehydrated in increasing grades of ethanol and xylene using a Tissue-Tek VIP (Miles Scientific). The sample was then embedded in PMMA and cut down with a bandsaw, sectioned, and ground to an optical finish using protocols previously described (Williams et al., 2025, 2012a, b). The bone under the simulated fracture fixation plate was analysed using backscattered electron imaging to examine the bone response and calculate the percentage of woven bone. The sections were adhered to plastic slides, then they were further ground and polished to ∼ 75 µ m for fluorescence microscopy to visualize the calcein green label and determine the percentage of reactive bone. Sections were lastly stained with Sanderson's rapid bone stain for qualitative light microscopy analysis. The number of osteoclasts in each section was counted, as osteoclast count correlates with infection severity (Croes et al., 2019).

### 2.10 Statistical methods

Statistical analysis and processing were performed using the Stata Statistical Software: Release 18 (StataCorp LLC, College Station, TX, USA). Error bars represent ± standard error unless otherwise noted.

Microbiological analysis compared the mean log_10_ reductions in CFUs (normalized to sample or by weight) between the treatment groups. The control plate CFUs were averaged for each animal, and a log_10_ transformation was applied to provide a baseline inoculum. Recovered samples that did not grow any CFUs were reported at the limit of detection (LOD) by artificially adding one colony to the least diluted plate. The normalized CFUs for each sample were summed for each animal, and the log_10_ transformation was applied to determine the total recovered bioburden. The mean log_10_ reduction was calculated as the difference between the log_10_-transformed control plate doubled CFUs (to account for two inocula plates) and the total sample CFUs. Positive log_10_ reductions indicated bacterial bioburden was reduced from the initial inoculum. Group means were tabulated and compared using a two-sample t test. A p value < 0.05 was considered statistically significant. Comparisons between individual sample bioburden and inocula were similarly compared with two-sample t tests, where a p value < 0.05 was considered statistically significant.

Reactive (fluorescent microscopy) and woven bone (backscattered electron imaging) analyses were also compared using two-sample t tests. Group means were tabulated and compared, with a p value < 0.05 used to indicate statistical significance. Osteoclast and μ -CT analyses used multiple locations, which introduced data clustering. A mixed-effect linear regression (multilevel model) accounted for nested locations within each sheep. The experimental condition (group) was a fixed effect, and the sheep was a random effect. Analyses were based on the t statistic due to the experimental sample size.

## 3 Results

### 3.1 Biofilm growth

Robust biofilms of 8.68 ± 0.88 log_10_ CFU for *S. aureus* and 9.05 ± 0.31 log_10_ CFU for *P. aeruginosa* reliably grew on inocula fixation plates. Representative scanning electron microscopy (SEM) images of biofilms on simulated fracture fixation plates were published previously (Kay et al., 2022). Outcomes suggested that all animals were inoculated with mature biofilms at the time of surgery.

### 3.2 Microbiological outcomes

**Figure 3:**
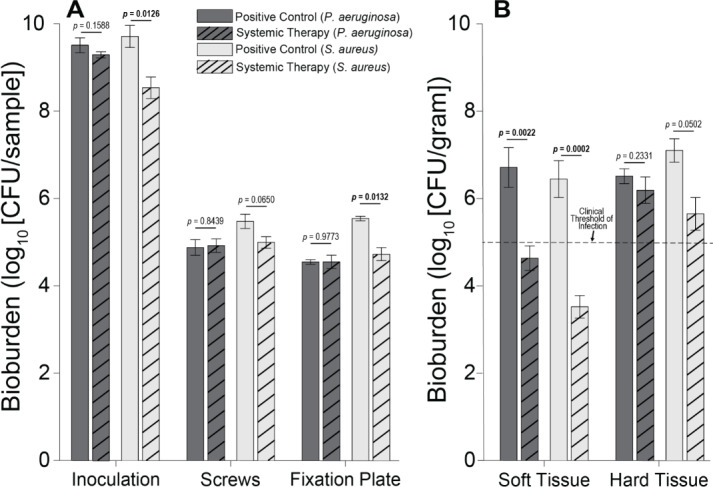
Individual sample bioburden. Average bioburden (log_10_ units) recovered, broken down by sample. **(A)** Hardware samples reported as the total bioburden collected per sample or set of samples (the four screw samples are aggregated). **(B)** Tissue samples normalized by weight. 5log_10_ is a commonly cited clinical threshold for infection. Statistical comparisons between the samples are reported in Table 2. Samples for positive controls are n=4 (groups 1 and 2), and samples for systemic therapy are n=8 (groups 3 and 4). Error bars represent ± standard error.

Microbiological outcomes and subdermal tissue observations at necropsy suggested that, despite suture lines showing signs of healing, all positive control of infection animals, and all those treated with antibiotics, had infected sites. There was a statistically significant difference in initial *S. aureus* inocula values between positive control of infection animals and those treated with systemic antibiotics ( p=0.0126 ; Table 2), yet animals were inoculated with $>8{\mathrm{log}}_{10}$ CFU, which was well above an infectious dose (Fig. 3).

**Figure 4:**
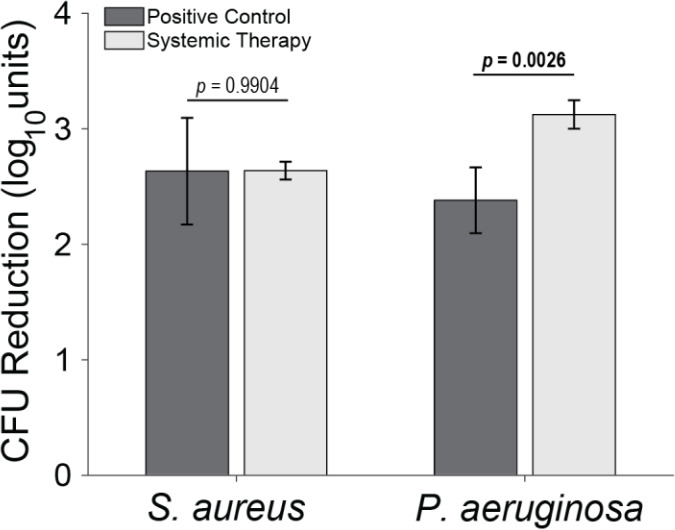
Total log_10_ reductions. The average group total bioburden reduction (log_10_ units) is reported as the difference between the inoculum and the total aggregated bacteria recovered in the nine samples. Statistical comparisons between the samples are reported in Table 2. Samples for positive controls are n=4 (groups 1 and 2), and samples for systemic therapy are n=8 (groups 3 and 4). Error bars represent ± standard error.

**Table 2:** Microbiology statistical comparisons. Comparisons are between the positive control group and the systemic therapy group. Statistically significant comparisons ( p<0.05 ) are bold.

*S. aureus*		*P. aeruginosa*	
Sample	p value	Sample	p value
Total log_10_ reduction	0.9904	Total log_10_ reduction	**0.0026**
Inoculation bioburden	**0.0126**	Inoculation bioburden	0.1588
Screws	0.0650	Screws	0.8439
Plate	**0.0132**	Plate	0.9773
Soft tissue	**0.0002**	Soft tissue	**0.0022**
Hard tissue	0.0502	Hard tissue	0.2331

*S. aureus*-inoculated animals treated with cefazolin and rifampin (Group 3) did not have a significant difference in total bioburden compared to positive controls ( p=0.9904 ; Table 2 and Fig. 4). The antibiotic therapy was less effective at reducing *S. aureus* bioburden in avascular or more inaccessible spaces, such as the screw threads and hard tissue (bone and screw tracks). In these particular areas, therapy failed to achieve statistical significance compared to the positive control ( p=0.0650 and p=0.0502 ; Table 2 and Fig. 3). Conversely, antibiotic therapy significantly outperformed the positive controls in more superficial locations, including the fixation plate and soft tissue ( p=0.0132 and p=0.0002 , respectively; Table 2 and Fig. 3).

Initial inocula baselines in animals inoculated with *P. aeruginosa* that served as positive controls or that received cefepime and rifampin had similar bioburden ( p=0.1588 ; Table 2 and Fig. 3). When pooled, *P. aeruginosa* bioburden was reduced significantly more in animals treated with antibiotics than in positive controls ( p=0.0026 ; Table 2 and Fig. 4). However, similarly to the *S. aureus *outcomes, systemic therapy was limited in less accessible regions, such as hard tissue and screws; bioburden levels remained similar to the positive controls ( p=0.2331 and p=0.8439 ; Table 2 and Fig. 3). Additionally, bioburden levels were nearly identical on the fixation plate between the positive control and antibiotic therapy groups ( p=0.9773 ; Table 2 and Fig. 3). Onboarding antibiotics demonstrated efficacy against *P. aeruginosa* in the soft tissue, where bacterial counts were nearly $3{\mathrm{log}}_{10}$ lower than in positive controls ( p=0.0022 ; Table 2 and Fig. 3). Despite this, cefepime and rifampin failed to reduce bacterial bioburden in hard tissue below the 10^5^ CFU g^−1^ of tissue infection benchmark (6.20 log_10_ units).

### 3.3 External sheep group inclusion

**Figure 5:**
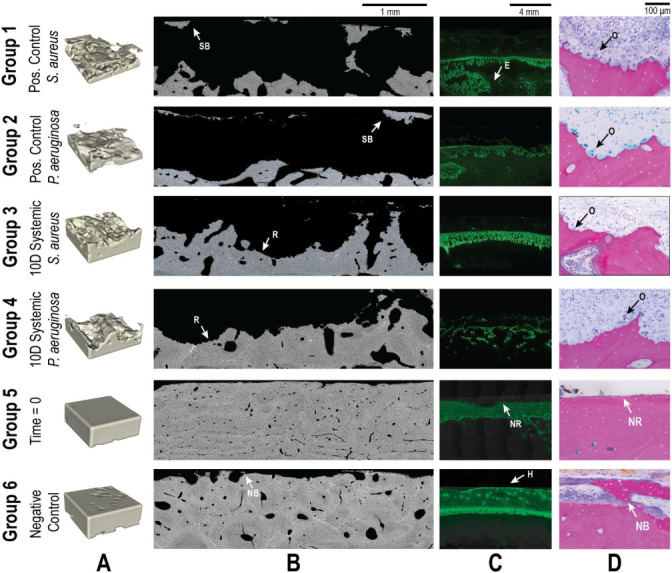
Representative qualitative histological outcomes. Representative images are shown for all groups (groups 1–6). Groups 5 and 6 were added from a previously published investigation. **(A)** μ -CT images demonstrating the bone volume in a 4 × 4 × 1.5 mm segmented area. Animals inoculated with bacteria (groups 1–4) demonstrated significant reductions in bone volume that were only partially attenuated by systemic antibiotics. **(B)** SEM images showed the bone response to treatment. Identified regions of sequestrum bone (SB), new bone (NB) growth, and resorption (R) are indicated with labelled arrows. **(C)** Fluorescent microscopy of the calcein green label showed bone remodelling. Areas of healthy remodelling (H), reactive bone in the endosteal (E) region, and no response (NR) were observed. **(D)** Light microscopy demonstrated significant osteoclast activity (O) in all animals inoculated with bacteria. At baseline (Group 5), no response (NR) was observed, and uninfected animals (Group 6) displayed new bone formation (NB).

**Figure 6:**
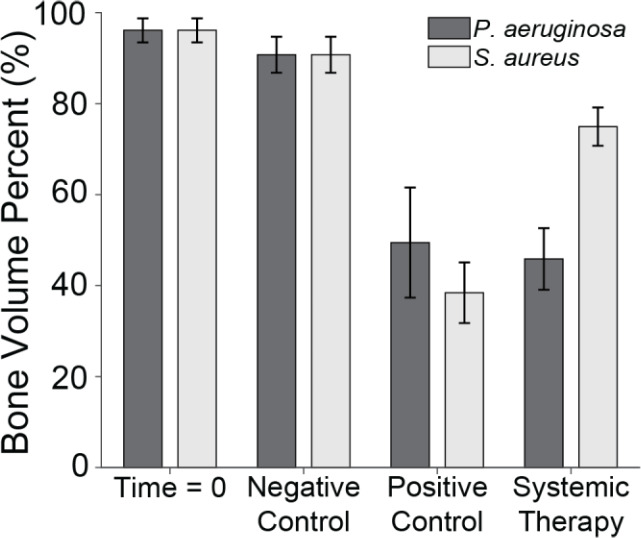
Bone volume percentage. The percentage of remaining bone volume under two sections of the proximal fixation plate was determined using μ -CT. Group means are displayed and statistical comparisons are reported in Tables 3 and 4. Samples are n=4 at time = 0 (Group 5), n=10 for the negative control (Group 6), n=8 for the positive control (groups 1 and 2), and n=10 for the systemic therapy (groups 3 and 4), with two samples per animal analysed. Error bars represent ± standard error.

**Figure 7:**
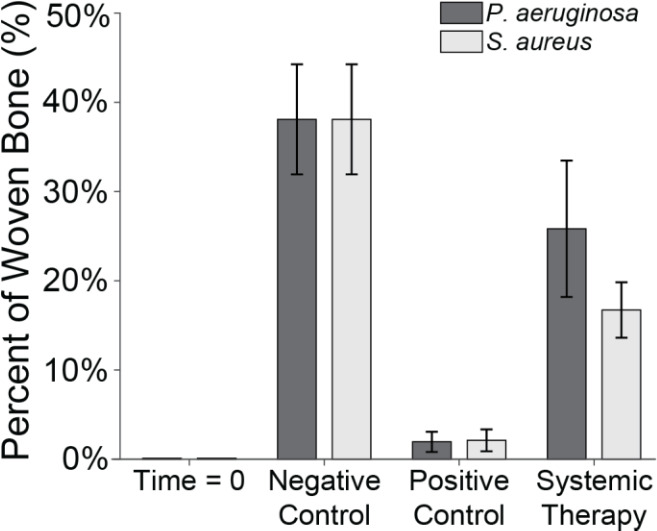
Woven bone percentage. The percentage of woven bone under a section of the proximal fixation plate was determined using SEM. Group means are displayed and statistical comparisons are reported in Tables 3 and 4. Samples are n=2 at time = 0 (Group 5), n=8 for the negative control (Group 6), n=4 for the positive control (groups 1 and 2), and n=8 for systemic therapy (groups 3 and 4). Error bars represent ± standard error.

**Figure 8:**
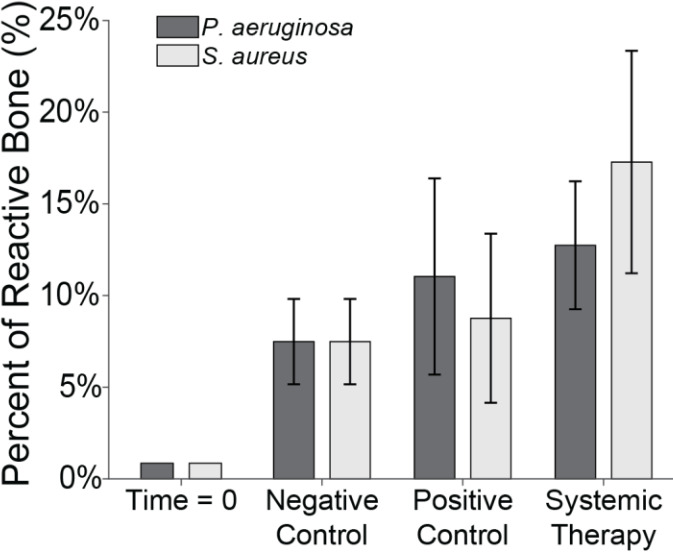
Reactive bone percentage. The percentage of reactive bone under a section of the proximal fixation plate was determined using fluorescent labelling techniques. Group means are displayed and statistical comparisons are reported in Tables 3 and 4. Samples are n=2 at time = 0 (Group 5), n=8 for the negative control (Group 6), n=4 for the positive control (groups 1 and 2), and n=8 for systemic therapy (groups 3 and 4). Error bars represent ± standard error.

**Figure 9:**
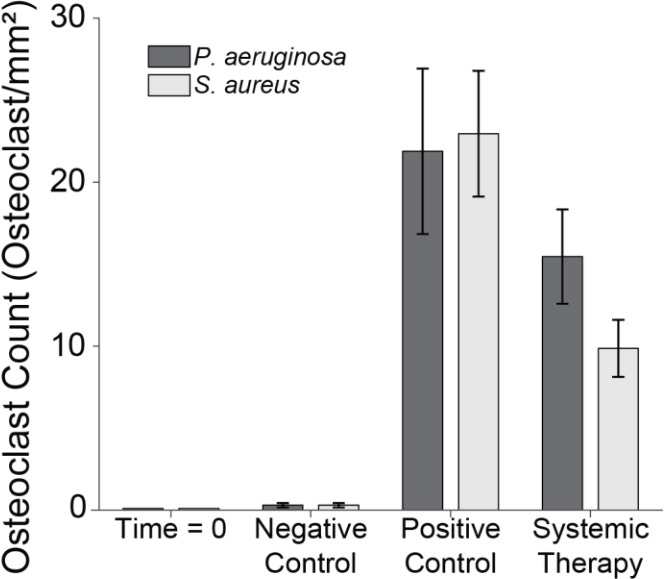
Osteoclast count. The number of osteoclasts under two sections of the proximal fixation plate was determined using light microscopy. Group means are displayed and statistical comparisons are reported in Tables 3 and 4. Samples are n=4 at time = 0 (Group 5), n=16 for the negative control (Group 6), n=8 for the positive control (groups 1 and 2), and n=16 for systemic therapy (groups 3 and 4), with two samples per animal analysed. Error bars represent ± standard error.

Two sheep groups that were worked on in a previously published study were included in the datasets of the sections below for comparison (Williams et al., 2025). The groups included animals sacrificed at time = 0 (immediately after surgery; Group 5) and a negative control group that received no bacteria (21 d endpoint; Group 6). These animals received the same surgery and histological processing described in the methods of this study but without the introduction of bacteria or antibiotic therapy. The histological data for each group are reproduced in Figs. 6–9 for both *S. aureus* and *P. aeruginosa* to allow visual and statistical comparison. A representative histological image can be seen in Fig. 5.

### 3.4 μ -CT

μ -CT was used to assess the percentage of remaining bone volume beneath the proximal fracture fixation plate (Fig. 6). Previously published data showed that animals displayed a high bone volume (96.1 % ± 2.6 %) at baseline (time = 0; Group 5), which was largely preserved in the negative control group after 21 d (90.7 % ± 4.0 %; Group 6) (Williams et al., 2025). The slightly lower bone volume in negative controls compared to baseline was attributable to the periosteum removal during surgery and natural bone porosity.

*S. aureus* infection significantly reduced bone volume. Specifically, positive control animals (Group 1) retained only 38.5 % ± 6.7 % of the bone beneath the proximal fixation plate, a statistically significant reduction compared to negative controls ( p=0.0000 ; Table 3: 1 vs. 6). This confirmed that infection successfully developed in positive controls. However, onboarding cefazolin and rifampin mitigated bone loss; Group 3 animals retained 74.9 % ± 4.2 % of bone. This was statistically significant compared to positive controls ( p=0.0020 ; Table 3: 1 vs. 3) and approached, but did not quite reach, statistical significance with negative controls ( p=0.0570 ; Table 3: 3 vs. 6).

**Table 3:** *S. aureus* histology statistical comparisons. Groups 1–4 correspond to Table 1. Group 5 represents the time = 0 animals, and Group 6 represents the negative control animals. Statistically significant comparisons ( p<0.05 ) are bold.

Fluorescent label		μ -CT		Osteoclast count		SEM	
Group comparison	p value	Group comparison	p value	Group comparison	p value	Group comparison	p value
1 vs. 6	0.7867	1 vs. 6	**0.0000**	1 vs. 6	**0.0000**	1 vs. 6	**0.0025**
3 vs. 6	0.1538	3 vs. 6	0.0570	3 vs. 6	**0.0030**	3 vs. 6	**0.0079**
5 vs. 6	0.2108	5 vs. 6	0.6060	5 vs. 6	0.9490	5 vs. 6	**0.0183**
1 vs. 3	0.3818	1 vs. 3	**0.0020**	1 vs. 3	**0.0010**	1 vs. 3	**0.0096**
1 vs. 5	0.3174	1 vs. 5	**0.0000**	1 vs. 5	**0.0000**	1 vs. 5	0.3140
3 vs. 5	0.2313	3 vs. 5	0.0540	3 vs. 5	**0.0420**	3 vs. 5	**0.0328**

Similarly, *P. aeruginosa* infection led to significant bone loss. Positive control animals (Group 2) retained only 49.5 % ± 12.1 % of bone, a statistically significant reduction compared to the negative control ( p=0.0010 ; Table 4: 2 vs. 6). However, unlike its efficacy against *S. aureus*, antibiotic therapy was far less effective against *P. aeruginosa*. Animals treated with cefepime and rifampin (Group 4) retained just 45.9 % ± 6.8 % of bone, which remained significantly lower than the negative control ( p=0.0000 ; Table 4: 4 vs. 6) and was not significantly different from positive controls ( p=0.7480 ; Table 4: 2 vs. 4).

**Table 4:** *P. aeruginosa* histology statistical comparisons. Groups 1–4 correspond to Table 1. Group 5 represents the time = 0 animals, and Group 6 represents the negative control animals. Statistically significant comparisons ( p<0.05 ) are bold.

Fluorescent label		μ -CT		Osteoclast count		SEM	
Group comparison	p value	Group comparison	p value	Group comparison	p value	Group comparison	p value
2 vs. 6	0.4876	2 vs. 6	**0.0010**	2 vs. 6	**0.0000**	2 vs. 6	**0.0024**
4 vs. 6	0.2310	4 vs. 6	**0.0000**	4 vs. 6	**0.0000**	4 vs. 6	0.2321
5 vs. 6	0.2108	5 vs. 6	0.7010	5 vs. 6	0.9540	5 vs. 6	**0.0183**
2 vs. 4	0.7892	2 vs. 4	0.7480	2 vs. 4	0.1150	2 vs. 4	0.0570
2 vs. 5	0.2736	2 vs. 5	**0.0040**	2 vs. 5	**0.0000**	2 vs. 5	0.3142
4 vs. 5	0.1422	4 vs. 5	**0.0020**	4 vs. 5	**0.0050**	4 vs. 5	0.1449

### 3.5 SEM

SEM was used to assess the percentage of woven bone as an indicator of new, healthy bone remodelling (Fig. 7). Previously published data showed that at baseline, no woven, i.e. healing, bone was observed (Williams et al., 2025). This was anticipated, since no time had elapsed for a healing response to occur. In negative control animals, substantial new bone formation (38.1 % ± 6.2 %) was observed in surgically impacted areas, indicating a natural healing response to the surgical procedure, which included periosteal stripping, simulated fracture placement, and fixation plate attachment. This suggested that the surgical model did not impair the animal's natural bone healing capacity.

In contrast, healing was severely attenuated in positive control animals (groups 1 and 3). Positive control animals inoculated with *S. aureus* (Group 1) exhibited significantly less woven bone (1.9 % ± 1.1 %) and increased bone resorption, consistent with infection development. This decrease was statistically significant versus the negative control ( p=0.0025 ; Table 3: 1 vs. 6). A similar response was observed in animals inoculated with *P. aeruginosa*, where new woven bone formation (2.1 % ± 1.2 %) was also significantly less compared to the negative control ( p=0.0024 ; Table 4: 2 vs. 6).

Systemic therapy moderately improved woven bone formation. Animals treated with cefazolin and rifampin had significantly more new woven bone growth (16.7 % ± 3.1 %) compared to the positive control animals ( p=0.0096 ; Table 3: 1 vs. 3), though significantly less than negative controls ( p=0.0079 ; Table 3: 3 vs. 6). A similar trend was observed in animals with *P. aeruginosa* infection: more woven bone formation was observed when cefepime and rifampin (Group 4) were onboarded (25.8 % ± 7.6 %) than in positive controls. However, due to increased variability in the response, bone formation was not statistically different from the negative or positive controls ( p=0.2321 and p=0.0570 ; Table 4: 4 vs. 6 and 2 vs. 4).

### 3.6 Fluorescent labelling

Fluorescent labelling was used to quantify reactive bone growth as an indicator of unhealthy bone formation (Fig. 8). Animals at baseline (time = 0) had minimal reactive bone under the proximal fracture fixation plate (0.85 %). The negative control animals that only underwent the surgical procedure showed a mild increase in reactive bone (7.5 % ± 2.3 %) (Williams et al., 2025). 

Infection with *S. aureus* elevated reactive bone formation. Positive controls (Group 1) displayed a modest increase in bone formation (8.8 % ± 4.6 %) that was not statistically significant versus negative control animals ( p=0.7867 ; Table 3: 1 vs. 6). Surprisingly, cefazolin and rifampin therapy (Group 3) exacerbated the response (17.3 % ± 6.1 %), though it remained statistically insignificant versus negative controls ( p=0.1538 ; Table 3: 3 vs. 6).

A similar trend was seen in *P. aeruginosa*-infected animals. Positive control animals exhibited a slight increase in reactive bone (11.0 % ± 5.3 %), which was elevated by cefepime and rifampin (12.7 % ± 3.5 %). Like with *S. aureus*, neither displayed significance versus the negative control animals ( p=0.4876 and p=0.2310 ; Table 4: 2 vs. 6 and 4 vs. 6).

### 3.7 Light microscopy

Light microscopy was used to enumerate osteoclast count (osteoclasts mm^−2^), a known indicator of infection severity (Fig. 9) (Croes et al., 2019). Negligible osteoclast activity was observed at baseline (0.1 mm^−2^) and remained low in negative control animals (0.3 ± 0.1 mm^−2^).

Infection significantly increased osteoclast activity. In positive control animals, osteoclast counts rose sharply in both *S. aureus* (22.9 ± 3.8 mm^−2^) and *P. aeruginosa* (21.9 ± 5.0 mm^−2^) infections versus negative controls ( p=0.0000 and p=0.0000 ; Tables 3 and 4: 1 vs. 6 and 2 vs. 6). Systemic therapy had varying effects. *S. aureus* animals treated with cefazolin and rifampin (9.9 ± 1.7 mm^−2^) demonstrated significantly reduced osteoclast activity compared to positive controls ( p=0.0010 ; Table 3: 1 vs. 3) but remained elevated relative to negative controls ( p=0.0030 ; Table 3: 3 vs. 6). However, in *P. aeruginosa*-infected animals (15.5 ± 2.9 mm^−2^), cefepime and rifampin treatment did not significantly reduce osteoclast counts versus positive controls ( p=0.1150 ; Table 4: 2 vs. 4) and levels remained higher than in negative control animals ( p=0.0000 ; Table 4: 4 vs. 6).

## 4 Discussion

Biofilm-related infections remain a major complication in traumatic injury treatment, particularly when orthopaedic hardware is involved. This study highlighted the potential limitations of systemic antibiotic therapy in controlling biofilm-associated infections in an established ovine model of FRI. While some reduction in infection markers was observed, such as partial decreases in bacterial burden and signs of bone remodelling, these improvements were insufficient to meet clinically significant thresholds for infection resolution and generally not statistically significant versus positive control animals. Biofilms of *S. aureus* and *P. aeruginosa* persisted above the $10^{5}$ CFU g^−1^ threshold, and histological analysis continued to show features of active infection and negative bone response. These findings suggest that although 10 d of systemic therapy initiated some degree of infection control, it failed to fully eradicate biofilm and will likely lead to recurring infection. This work supported our hypothesis and further validated the model as a robust platform for studying biofilm persistence and therapeutic response in the context of FRI.

Systemic antibiotic therapy has remained a cornerstone of infection management in FRIs for over 50 years (Gustilo and Anderson, 1976; Patel et al., 2023). Despite this, few new antibiotics have been discovered, with lipopeptides, the most recent new class, identified in the 1980s. Thus, surgeons and research efforts focus on alternative drug delivery strategies, such as local antibiotic applications: powders, beads, hydrogels, or molecular carriers (Boot et al., 2022; Steadman et al., 2023; Williams et al., 2025). However, even these approaches are failing to make meaningful headway, and systemic therapy remains the first and primary line of defence in FRI cases. A secondary analysis of the fluid lavage of open wounds (FLOW) cohort found that 10 % of open fracture infections were unresolved 1 year after management surgery (Prada et al., 2021). The Oral versus Intravenous Antibiotics for Bone and Joint Infection (OVIVA) trial with 1054 patients similarly demonstrated 1-year definitive treatment failure rates of 14.6 % and 13.2 % for patients treated with systemic intravenous and oral antibiotics, respectively (Li et al., 2019). Delivering drugs systemically is also inherently limited in FRIs due to compromised vasculature, which restricts antibiotic diffusion, and systemic dilution, which prevents achieving the high local concentrations needed to disrupt biofilms. Appropriate animal models are essential to evaluate emerging treatment strategies against current standards of care.

A 2023 international consensus meeting on musculoskeletal infection highlighted the lack of standardized animal models for infection studies (Jennings et al., 2024). A strong consensus determined that no single timeline or species is ideal for studying infection resolution. Smaller animal models, while widely used, may lack clinical relevance due to differences in skeletal structure, bone healing, and antibiotic pharmacokinetics. Ovine models provide a more clinically translatable system due to their larger body size, which allows dosing with clinically relevant antibiotic quantities and a skeletal structure more comparable to humans (Roux et al., 2021). Despite these advantages, limitations in ovine models still exist.

A 2024 review found that 88 % of ovine orthopaedic infection studies used planktonic bacteria for inoculation (Beagan et al., 2024). Only a small number of studies involved biofilm inoculation or delayed antibiotic treatment to allow a mature biofilm to develop (Boot et al., 2021, 2022; Williams et al., 2025). This is a critical limitation, as mature biofilms are far more tolerant to systemic therapy than planktonic bacteria. The present study underscored these limitations: systemic therapy alone failed to significantly reduce *S. aureus* biofilm burden compared to positive controls and produced only a modest ( ∼1 log_10_) reduction in *P. aeruginosa* infection. Notably, bacterial counts in the hard tissues of animals treated with antibiotic therapy remained above the clinical benchmark for infection resolution (10^5^ CFU per gram of tissue) (Raahave et al., 1986; Robson and Heggers, 1969). Although systemic therapy achieved measurable reductions in soft tissue bacterial load, significant residual bioburden on orthopaedic hardware and within hard tissues will likely drive ongoing infection. *P. aeruginosa* bioburden recovered from animals treated with systemic antibiotics, for example, was nearly identical to untreated positive controls of infection. Many animal models with healthy subjects also use an even more rigorous 10^2^–10^3^ CFU limit to quantify successful infection treatment (Jennings et al., 2024). All recovered hardware and tissue sample averages in this study exceeded that standard. These results continue to support the rigour of this experimental ovine model, which has been in development for over 1 decade (Williams et al., 2012a, b). Previous studies using this model with MRSA biofilms yielded similar findings, with systemic vancomycin failing to reduce bacterial loads below 10^5^ CFU per gram of tissue (Williams et al., 2025).

Few ovine infection studies using mature biofilms exist that examine histological outcomes of systemic therapy and are often only used to evaluate a binary outcome of infection (infection present or not) (Boot et al., 2021). Despite this, the histological impacts of FRI are well characterized. Infection disrupts bone homeostasis, increasing osteoclast activity, inhibiting woven bone formation, and promoting reactive bone deposition (Croes et al., 2019; Lee et al., 2016). Many of these trends were evident in this study: infected animals exhibited decreased total bone volume and woven bone percentage, along with increased osteoclast activity and reactive bone formation. While systemic antibiotic therapy generally improved these parameters, the changes were typically not statistically significant. Notably, systemic therapy demonstrated several encouraging outcomes. In *S. aureus* infections, treated animals retained significantly more bone compared to positive controls, a finding that aligned with a significant reduction in osteoclast count. Systemic therapy also promoted significantly greater new bone formation in both *S. aureus* and *P. aeruginosa* infections, suggesting that infection was suppressed enough to permit the onset of bone healing. Still, the substantial residual bioburden observed suggests that systemic antibiotics may have only delayed, rather than resolved, infection. This raises the possibility that disease progression would have continued beyond the 21 d study period. Further, systemically treated animals showed an increase in reactive bone compared to positive controls. This result was counterintuitive because of a generally positive correlation between CFUs and reactive bone formation (Croes et al., 2019). Severe infection may exceed a threshold where inflammatory signalling becomes inhibitory rather than stimulatory to bone formation. Antibiotic treatment may have reduced infection severity below this inhibitory threshold, permitting reactive bone formation even when bacterial clearance was incomplete. Another investigation demonstrated that DNA released from bacterial cells killed by antibiotics results in increased inflammation, even compared to untreated animals (Gross et al., 2024). While the results in the study likely have multifactorial underpinnings, several potential mechanisms exist. These results also align with previous studies using this model, which found only mild histological improvements with systemic therapy (Williams et al., 2025). Future investigations will evaluate whether local therapies can provide greater histological benefits.

This study had several limitations. We did not collect systemic antibiotic concentration data, which could be valuable for clinical translation. The rationale was that systemic blood concentrations for intramuscular cephalosporins are already well characterized clinically. These drugs are well tolerated and bioavailable, and they rapidly reach safe systemic concentrations after transient post-injection spikes without notable accumulation (Barbhaiya et al., 1990; Kusaba, 2009). Additionally, systemic absorption of intramuscular cephalosporins and oral rifampin has been demonstrated in previous ovine studies (Ismail, 2005; Jernigan et al., 1991). Nevertheless, bioavailability, especially for oral antibiotics, may be reduced due to differences between human and ruminant digestive tracts.

Another limitation was the duration of antibiotic therapy. Animals received 10 d of systemic antibiotics, after which they survived for an additional 11 d. This duration is shorter than clinical treatment timelines, which typically extend for several weeks and may continue for months in difficult, recalcitrant infections (Prada et al., 2020). In this model, the shortened therapy may have allowed bacterial persistence or regrowth, and infection may have been resolved if therapy had been continued. This design was intentional, to capture infection dynamics following a defined course of systemic therapy, rather than to model complete clinical resolution. Given the well-documented resilience of biofilms and their ability to tolerate therapeutic concentrations of antibiotics, we aimed to evaluate whether bacteria seeded as mature biofilms could withstand a standard treatment regimen and assess microbiological and histological outcomes at a later time point. This strategy also allowed direct comparison with our previous work evaluating systemic vancomycin against MRSA biofilms in the same model (Williams et al., 2025). By establishing the performance of systemic therapy after 10 d of treatment versus *S. aureus* and *P. aeruginosa*, we develop the foundation for a clinically relevant preclinical platform for testing novel antimicrobial strategies that may address the limitations of systemic therapy. The ovine model employed offers translational advantages, including the ability to accommodate human-scale medical devices and delivery systems. This positions the model as a valuable tool for evaluating emerging therapeutic approaches such as localized antibiotic delivery, antimicrobial biomaterials, or other combination strategies that integrate surgical intervention with targeted antimicrobial technologies. By characterizing the baseline efficacy of standard systemic therapy, this work establishes a foundation for comparative assessment of novel interventions designed to manage FRIs.

Previous studies demonstrated superior outcomes with local therapy compared to systemic treatment alone against MRSA (Williams et al., 2025). Future studies will explore the performance of local therapies in this ovine model against *S. aureus* and *P. aeruginosa* biofilms. Evaluating whether these findings extend to other bacterial species will enhance our understanding of FRI. Additionally, separate studies that will be published soon that examine polymicrobial biofilms, which are responsible for approximately one-third of open fracture infections (Jorge et al., 2018). By continuing to investigate clinically relevant antibiotic therapies in this rigorous ovine model, we can better assess current standards of care and develop new technologies to improve FRI management.

## 5 Conclusions

This study highlighted the limitations of managing biofilm-related infections using systemic antibiotics alone. In an ovine model of simulated FRI, 10 d of systemic therapy was insufficient to reduce *S. aureus* and *P. aeruginosa* biofilms below the clinical definition of infection. While systemic antibiotics reduced bacterial loads in soft tissue, their impact on more inaccessible sites, such as bone and implant surfaces, was minimal. In a clinical setting, high remaining untreated bioburden would contribute to recurrent infection. Histological analysis similarly showed only modest improvements in bone healing compared to untreated animals. These findings highlight the need for innovative, targeted antibiotic delivery strategies to overcome the limitations of systemic therapy in managing biofilm-associated FRIs.

## Data Availability

The data used are confidential, as they pertain to the development of an intellectual property (IP) product.
